# Potentials of mono- and multi-metal ion removal from water with cotton stalks and date palm stone residuals

**DOI:** 10.1007/s11356-023-27137-4

**Published:** 2023-05-01

**Authors:** Heba Nagy, Manal Fawzy, Elsayed Hafez, Alaa El Din Mahmoud

**Affiliations:** 1 Environmental Sciences Department, Faculty of Science, Alexandria University, Alexandria, 21511 Egypt; 2 Green Technology Group, Faculty of Science, Alexandria University, Alexandria, 21511 Egypt; 3 National Biotechnology Network of Expertise (NBNE), Academy of Scientific Research and Technology (ASRT), Cairo, Egypt; 4 Plant Protection and Biomolecular Diagnosis, Arid Lands Cultivation Research Institute (ALCRI), City of Scientific Research and Technological Applications (SRTA-City), Alexandria, 21934 Egypt

**Keywords:** Cadmium/lead/zinc, Multi-metal adsorption, Models, Desorption, Real wastewater

## Abstract

In this work, cotton stalks (*Gossypium barbadense*) and date palm stones (*Phoenix dactylifera*) have been used as biosorbents to remove cadmium; Cd(II), lead; Pb(II), and zinc; Zn(II) from mono- and multi-solutions. Each biosorbent was characterized using SEM-EDX, and FT-IR. The findings showed that pH, dose, contact time, metal concentration, and particle size affect the treatment process. The adsorption pattern was Pb(II) > Cd(II) > Zn(II) for both biosorbents. The adsorption performance of cotton stalks was higher than that of date palm stones. The fitted maximum uptake capacities; *q*_m_ of cotton stalks were higher than those of date palm stones. The maximum adsorption at optimum conditions of Pb(II), Cd(II), and Zn(II) with cotton stalks were 98%, 92.1%, and 78.9%, respectively, within 30 min. While the maximum adsorption of Pb(II), Cd(II), and Zn(II) with date palm stones were 94.6%, 76%, and 68.6%, respectively. Results confirmed the antagonistic effect of heavy metal removal at optimum conditions. Biosorbents could remove ~ 100% of the metal ions from real wastewater samples. Regeneration investigation revealed a successful reusability of both biosorbents for four cycles.

**Supplementary information:**

The online version contains supplementary material available at 10.1007/s11356-023-27137-4.

## Introduction

Shortage and lacking freshwater are one of the main challenges in agriculture and food security fields. Out of 800 million people in North Africa, ~ 300 million reside in areas with limited access to water resources (Mayaux et al. 2022; Radingoana et al. 2020). Reusing agricultural drainage water can be an ideal solution to solve irrigation water insufficiency. Due to salinity issues, reused drainage water quality is extremely important, particularly in arid regions like Egypt (Ashour et al. 2021; Ezzat and Elkorashey 2020). Consequently, integrated water and wastewater management/reuse are needed urgently for sustainable practices. Wastewater treated to the proper quality can replenish water supplies and diminish the acute gap between demand and availability in view of climate change impact (Dotaniya et al. 2022; Mahmoud et al. 2021c). However, heavy metals are generated from a vast array of industrial activities and reach the aquatic ecosystems to be accumulated in aquatic habitats.

Heavy metals are dangerous even if they have been found in traces in real wastewater effluents. Heavy metals were frequently found, including cadmium, chromium, copper, lead, and zinc, in agriculture wastewater as used in manufacturing of pesticides and herbicides. Cadmium (Cd) and lead (Pb), the most frequent harmful heavy metals, are known as the “big three” heavy metals, along with mercury (Kahlon et al. 2018). Furthermore, zinc (Zn) is one of the most often found heavy metals in acid mine drainage and industrial effluents from galvanizing facilities (Pratap et al. 2022). Despite the fact that Zn is a necessary trace element for living organisms, it is poisonous to them at millimolar doses.

Hence, the removal of these elements from wastewater to be reused is still a challenge in view of high tertiary treatment cost. The majority of conventional wastewater treatment methods (including reverse osmosis, ion exchange, and precipitation) possess limitations in most industrial applications. This can be due to high capital with complexity to operate, low selectivity, and energy costs. Hence, they are limiting factors in water reuse.

Biosorption process can be the green technology for wastewater treatment. It has great effectiveness in sequestering dissolved metals from solutions with diluted concentrations. Because of its procedure over a wide range of temperature and pH without releasing secondary contaminants, biosorption is an appropriate technology for treating low-concentration and high-volume wastewaters (Sathya et al. 2022; Vijayaraghavan and Balasubramanian 2015). Many kinds of biomasses were used as biosorbents for heavy metal removal. For instance, citrus *Sinensis* peel (El Malti et al. 2022), bamboo sawdust/rice husk (Kwikima et al. 2022), rice straw (Ameen Hezam Saeed et al. 2022; Nasr et al. 2017), and orange peel (Priyadharshini and Abraham 2022) were applied for the elimination of cadmium (Cd). Wheat stem biomass (Jalali et al. 2021), common beans (*Phaseolus vulgaris*) (Salazar-Pinto et al. 2021), and rice straw (Xu et al. 2021) were tested for lead (Pb) adsorption. Other biomasses, such as lemon, watermelon, pineapple, and banana peels (Yılmaz and Tugrul 2021), were used for zinc elimination from water.

The regeneration possibility of the biosorbents is a critical step to evaluating their performances and feasibility in real wastewater applications. Effective water treatment and reuse applications can deliver environmental and socioeconomic benefits. Therefore, using locally available agricultural wastes such as date palm stones and cotton stalks as biosorbents possess dual benefits: minimizing biological waste and providing a cost-effective solution for water pollution.

It is worth noting that there is still an insufficient comparative study of various agricultural byproducts as biosorbents and few investigations on multi-metal solutions and real wastewater to simulate the actual conditions in the treatment process. The main goal of this work is to test the possibility of using locally available agricultural wastes for the tertiary treatment of wastewater to be reused for agricultural purposes. The objectives are to (1) evaluate the biosorption potentialities of cotton stalks and date palm stones for Cd(II), Pb(II), and Zn(II) in mono- and multi-metal solutions, (2) optimize the environmental factors controlling the biosorption process, (3) investigate the recyclability of the investigated biosorbents, and (4) evaluate the effectiveness of the investigated biosorbents using real wastewater.

## Materials and methods

### Preparation and characterization of biosorbents

Cotton stalks and date palm stones were collected from agricultural ecosystem of El-Mamoura area situated in the sub-urban of Alexandria City, Egypt. The collected samples were firstly dissected, cleaned, rinsed with tap and deionized water, and finally oven dried at 70 °C for 48 h. To obtain homogenous powders, plant samples were finely crushed and ground by a stainless-steel grinder (Moulinex,700 w, France) and then graded by a stainless-steel sieve with a mesh size (0.25, 0.5, and 1 mm) to obtain known particle sizes. The resultant powder was stored in plastic bottles for further use. The utilized characterization techniques are illustrated in the Supplementary information.

### Batch biosorption experiments

Based on the analysis results of the collected real wastewater, the stock solutions of the targeted metal ions were diluted with appropriate quantities of double-distilled water. 1N HCl/NaOH solutions were used to adjust the pH. Measurements of pH were done by pH meter (Martini combined meter, Mi805, Romania). Each experiment was carried out in triplicates at 25 ± 3 °C, and average values were taken for further calculations.

A specific biosorbent dose was applied to each flask of 30 mL of known Cd, Pb, and Zn solutions. The resulting suspension was continually stirred by the HAF incubator shaker. Teck for 120 min at a 150 rpm agitation rate. Once finished, a suspension sample was obtained and filtered using Whatman filter paper no. 1 to remove biosorbent particles.

By utilizing an atomic absorption spectrophotometer (Thermo Scientific SARIS A.A spectrometer), the filtrate’s residual metal concentrations were determined. The effects of contact time (1–120 min), pH (2–8), biosorbent doses (1.66–33.33 g L^−1^), metal concentrations (10–50 mg L^−1^), and particle size of plant biomass (0.25–1.0 mm) on targeted heavy metals were investigated.

Subsequent to the mono-metal ion removal, the multi-metal ion removal was conducted at the optimum pH values. To investigate the antagonistic effects of these metals on the biosorption process at the optimal pH and biosorbent dosage as illustrated in multi-metal adsorption section.

### Evaluation of biosorption experiments

The uptake capacity and removal percentage of Cd(II), Pb(II), or Zn(II) were calculated according to Eqs. (S1) and (S2) (Badr et al. 2020; Mahmoud et al. 2022b). Kinetics models were applied to assess the biosorption mechanism (Eqs. S3–S5). Different models describe the equilibrium between the adsorbate and biosorbent using Eqs. (S6)–(S8).

### Multi-metal adsorption experiments

The standard tertiary solutions of each metal were prepared in a concentration ratio of 1:1:1 and 1:2:3 with double-distilled water. Thirty milliliters of 30 mg L^−1^ multi-metal solution (10 ppm for each metal with a ratio of 1:1:1 and 5 ppm, 10 ppm, and 15 ppm for Cd(II), Pb(II), and Zn(II) with a ratio of 1:2:3) was added into each flask. The following conditions were maintained based on common optimum conditions for the studied metals that were detected from batch biosorption experiments.

### Real wastewater investigation

The wastewater samples were collected in triplicates from El-Ameya Drain, a mixed municipal, agricultural, and industrial drain located at El-Tabia, northeast coast of Alexandria, Egypt, along four different seasons during 2021–2022. The physicochemical characteristics and heavy metal contents were examined.

### Regeneration of biosorbents

To conduct the regeneration investigation of the studied biosorbents, a detailed procedure was described in the supplementary information.

## Results and discussion

### Characterization of the biosorbents

**Fig. 1:**
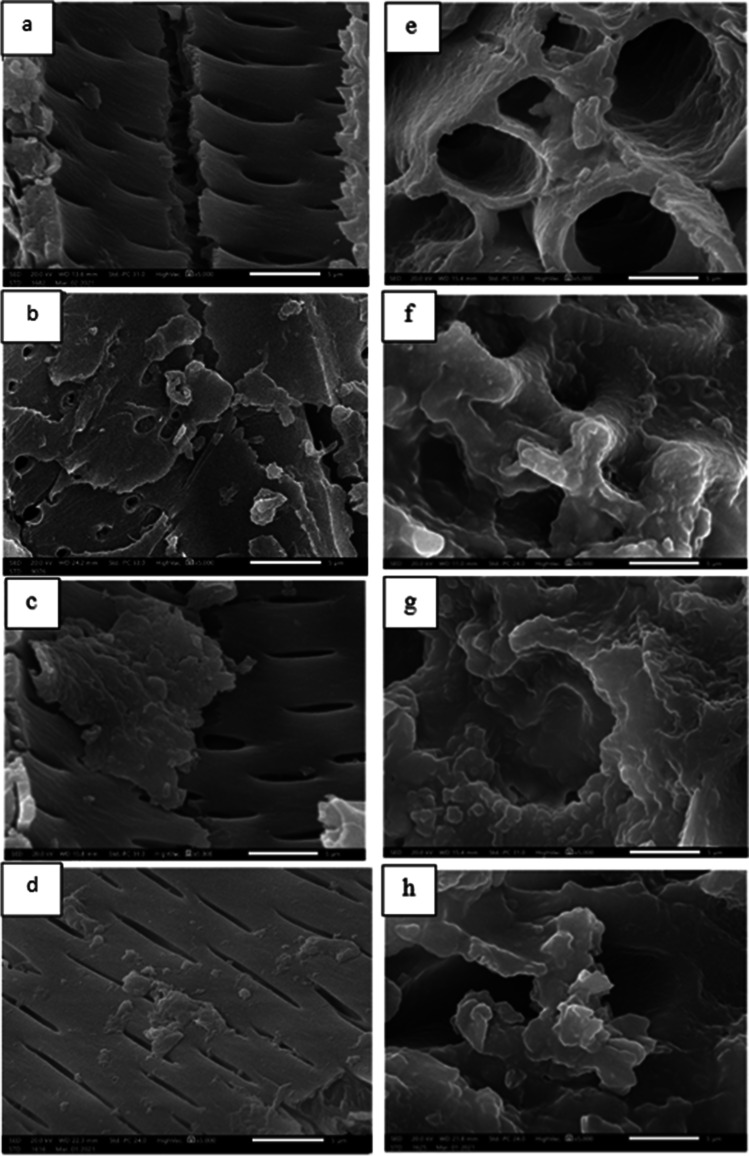
Photomicrographs showing surface morphology of the investigated **a** cotton stalk and **e** palm dates before and after **b**, **f** Cd(II) ions, **c**, **g** Pb(II) ions, and **d**, **h** Zn(II) ion biosorption (magnification × 5000 at 20 kV)

The surface morphology of the biosorbents of cotton stalks and palm date stones before and after biosorption as seen by SEM were given in Fig. 1a–h, respectively. The surface textures of the investigated biomass before the biosorption process were smooth and perforated. Subsequent to the biosorption process, the surface of the plant biomass becomes granulated, and all pores are covered.

**Fig. 2:**
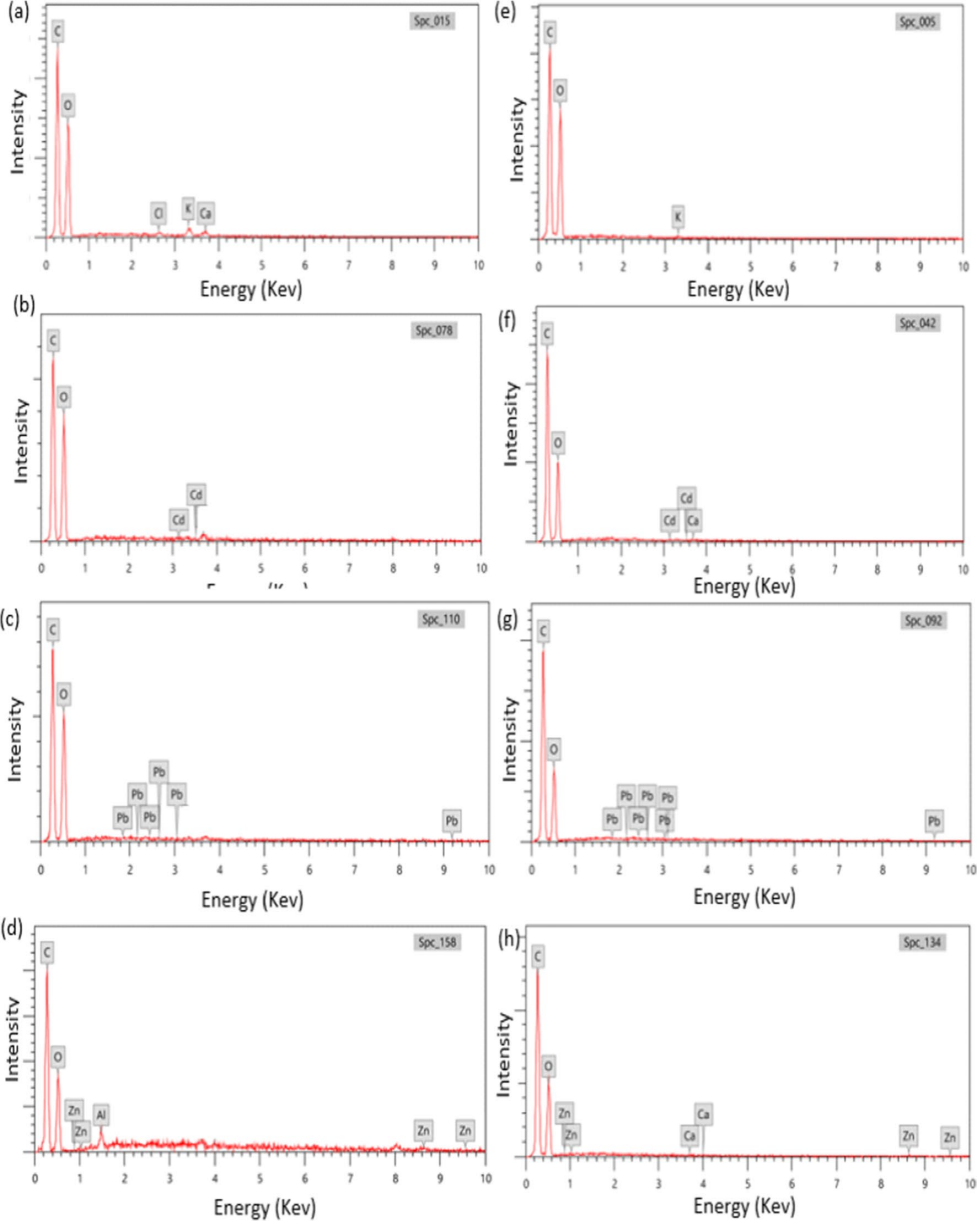
EDX spectra of the investigated **a** cotton stalk and **e** palm dates before and after **b**, **f** Cd(II) ions, **c**, **g** Pb(II) ions, and **d**, **h** Zn(II) ions biosorption

Furthermore, the relative concentration of elements of the investigated biosorbents before and after biosorption was depicted by EDX, as illustrated in Fig. 2. SEM analysis was performed for evaluating the fractions, flaws, and pollutants on the surface of the biosorbent. The relative concentrations of Cd(II), Pb(II), and Zn(II) on the surface of date palm stones were 0.44%, 0.44%, and 0.71%, respectively. While their relative concentrations on the surface of cotton stalks were 0.85%, 0.8%, and 2.1%, respectively. Also, potassium (K) and calcium (Ca) were detected on the raw cotton stalks and disappeared after the biosorption. Instead, heavy metal ion signals were detected, suggesting ion exchange mechanism (Pino et al. 2006; Zhang et al. 2023) as illustrated in Eqs. (1)–(3).

1 $$({R-\mathrm{COO})}_{2}\mathrm{Ca}+{\mathrm{Cd}}^{2+}\to ({\mathrm{R}-\mathrm{COO})}_{2}\mathrm{Cd}+{\mathrm{Ca}}^{2+},$$

2 $$(\mathrm{R}-\mathrm{COO})_2\mathrm{Ca}+\mathrm{Pb}^2+\to (\mathrm{R}-\mathrm{COO})_2\mathrm{Pb}+\mathrm{Ca}^{2+},$$

3 $$({R-\mathrm{COO})}_{2}\mathrm{Ca}+{\mathrm{Zn}}^{2+}\to ({\mathrm{R}-\mathrm{COO})}_{2}\mathrm{Zn}+{\mathrm{Ca}}^{2+},$$

**Fig. 3:**
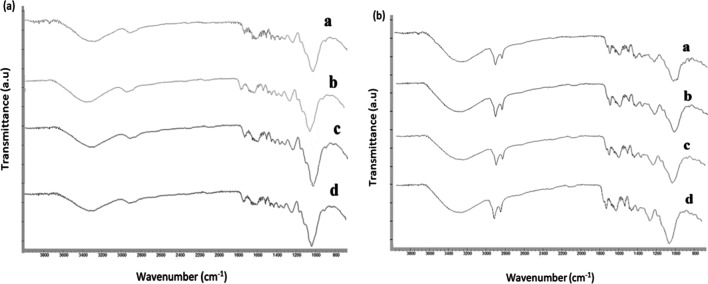
FTIR spectra of the investigated (a) cotton stalk and (b) date palm before (**a**) and after biosorption of **b** Cd(II), **c** Pb(II), and **d** Zn(II)

The presence of functional groups on the investigated biosorbents were provided by FT-IR in the range of ~ 4000–800 cm^−1 ^(Fig. 3a,b). A remarkable shift was recorded for hydroxyl group (–OH) stretching. The (–OH) peaks showed a shift from 3337.1 to 3324.7 (Cd(II)), 3328.9 (Pb(II)), and 3332.1 cm^−1^ (Zn(II)). The shift from 2917.2 to 2913.7 and 2917.2 cm^−1^ for Cd(II) and Pb(II), respectively, and disappeared for Zn (II) were assigned to aliphatic acid’s C–H stretching vibration (Subbaiah Munagapati et al. 2022).

A pronounced shift was recorded at 1733.7 cm^−1^, which shifted to 1734.2, 1733.5, and 1733.8 cm^−1^ for Cd(II), Pb(II), and Zn(II), respectively, corresponding to stretching vibration of C = O bond which found in carboxylic acids or esters (–COOH or –COOCH_3_). This shift associated with another shift from 1235.3 to 1234.5, 1235.3, and 1234.1 cm^−1^, which may assign to C–O stretching, this observation may confirm the presence of carboxylic ester. Shifted bands from 1644.9 to 16531.1 and 1653.3 for Pb(II) and Zn(II), respectively, were assigned to an asymmetric stretching of –COO– in ionic carboxylic group, while shifted bands from 1457.1 to 1457.6 (Cd(II)), 1457.3 (Pb(II)), and 1457.5 cm^−1^ (Zn(II)) were assigned to symmetric –COO– stretching in pectin (Iqbal et al. 2009). Table S1 summarizes the existing functional groups and the shifted ones for each investigated biosorbent.

### Biosorption experiments

#### Influence of contact time

**Fig. 4:**
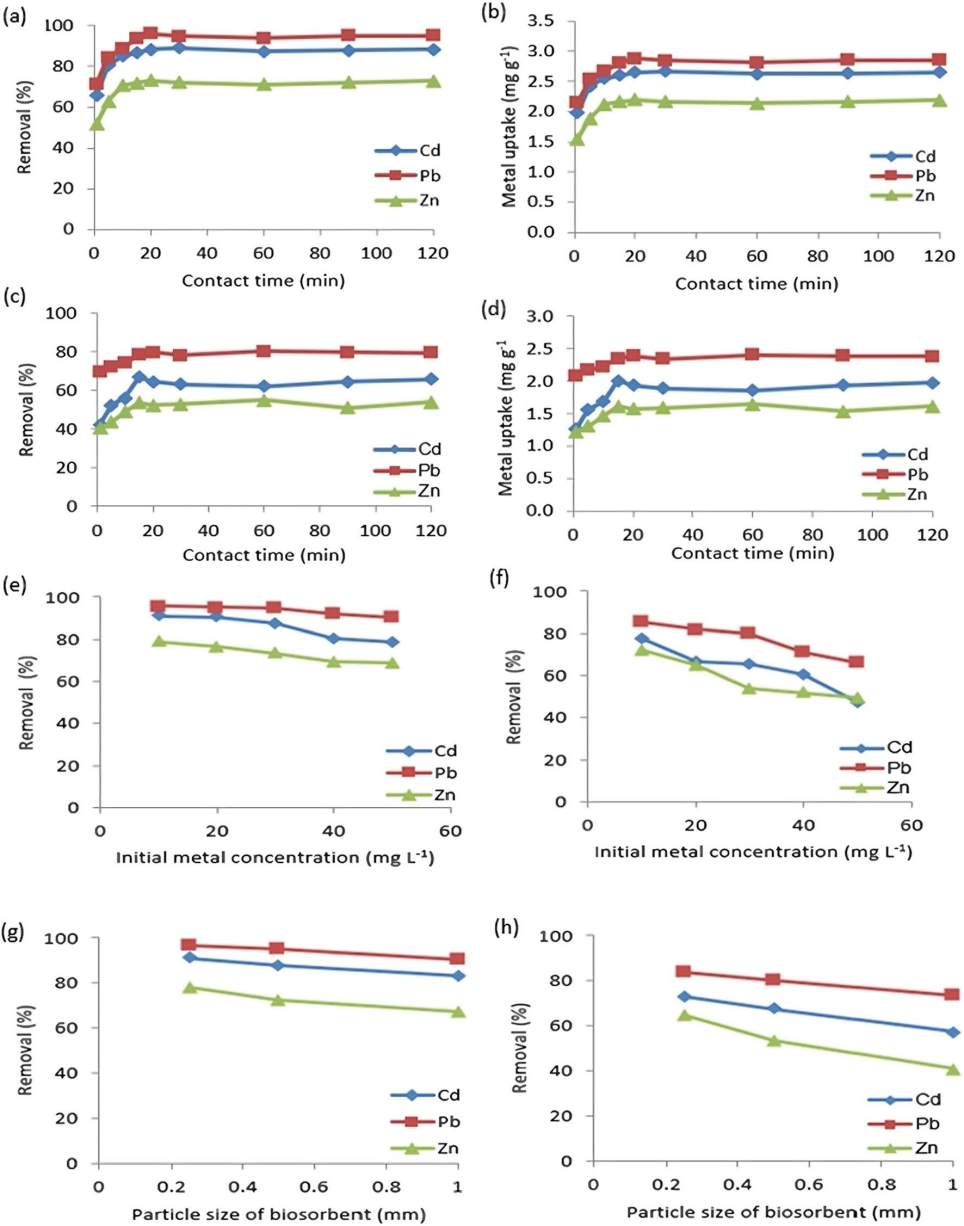
**a**–**d** Effect of contact time, **e**, **f** effect of metal initial concentration, and **g**, **h** effect of biosorbent particle size on Cd(II), Pb(II), and Zn(II) removal and uptake capacities for (a, b, e, g) cotton stalks and (c, d, f, h) palm dates

Figure 4a–d illustrates how contact time affects the removal and update capacities of Cd(II), Pb(II), and Zn(II) ions. It was found that % removal of the metal ions increased with increasing biosorption time until equilibrium is obtained. The studied metals were rapidly removed by cotton stalks and date palm stones in less than 20 min, and the equilibrium reached 60 min, compared to with sugarcane bagasse (Ezeonuegbu et al. 2021) and mango peel waste (Iqbal et al. 2009).

It is obvious that the removal of the metal’s ions was increased rapidly by the biosorbent because there were active binding sites on the biosorbent, and as these sites were gradually occupied, the sorption process became less effective in the later stages (El Din Mahmoud and Fawzy 2016). Similar behavior was attained during the first 15 min with ˃ 90% Pb(II) biosorption using cucumber peels, and the equilibrium was reached at 60 min (Basu et al. 2017). Recently, Fertu et al. (2022) could retain 66% Pb(II), 60% Cd(II), and 57% Zn(II) on soybean biomass in 30 min.

#### Influence of metal ion concentration

Metal ions are sorbed by specific active sites at low concentrations in the aqueous solutions. However, at greater concentrations, the saturation of biosorption sites results in a reduced biosorption yield (Fawzy et al. 2016; Mahmoud and Fawzy 2015; Mahmoud et al. 2016).

Although the increment of metal uptake capacity (*q*_e_) was observed, the removal (*R*%) was decreased, which may be explained by a lack of surface area to adapt more of the metal present in the solution. This happens as a result of an increment in the ions number contending for the available active sites on the surface of the biosorbents (Mahmoud et al. 2021b).

Applying cotton stalks as a biosorbent with different metal ion concentrations (Fig. 4e), Pb(II) and Cd(II) ions removal decreased from 95.48 and 91.02% to 90.34 and 78.64% with increasing the concentrations from 10 to 50 mg L^−1^. On another hand, Fig. 4f illustrates Pb(II) and Cd(II) ions removal with palm date stones decreased from 85.20% and 77.53% to 65.84% and 47.04%. While Zn(II) removal with cotton stalks decreased from 78.83 to 68.63% and decreased from 72.18 to 49.58% with palm date stones with increasing the initial concentration in the same range.

#### Influence of biosorbent particle size

Figure 4g and h shows the effect of different particle sizes of the investigated biosorbents on adsorption of Cd(II), Pb(II), and Zn(II) at 120 min. It is obvious that the heavy metals removal increased with decreasing biosorbent granular size. The adsorption removal of the studied metal ions was 1.1–1.2 higher than those of cotton stalks at ˃ 0.5 mm. While the removal of Pb(II), Cd(II), and Zn(II) were 1.14, 1.27, and 1.59 higher than those of date palm stones at ˃ 0.5 mm. Hence, the smaller the particle size of biosorbent, the greater its surface area, resultant increase in the binding sites for adsorption (Wang et al. 2022).

### Influence of pH

pH is a key aspect because it has an impact on how heavy metals are sequestered onto the surface of the biosorbent (Mahmoud 2020). As a result, the sorbent functional groups or the targeted elements in the water may have been ionized (Yang et al. 2016).

**Fig. 5:**
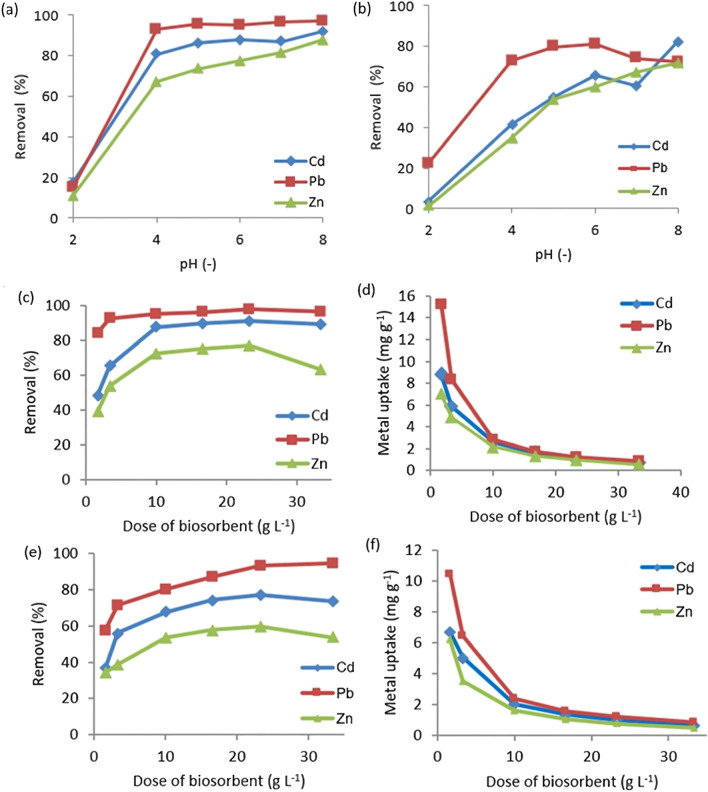
**a**, **b** pH influence on the adsorption process and **c**–**f** effect of the biosorbent doses on Cd(II), Pb(II), and Zn(II) removal and uptake capacities (a, c, e) cotton stalks and (b, d, f) palm dates [*C*_0_ = 30 mg L^−1^; biosorbent dose = 10 g L.^−1^; agitation speed = 150 rpm; temp. = 25 ± 3 °C]

The pH effects on the biosorption behavior of both biosorbents for the metal ions were evaluated within pH 2–8, as demonstrated in Fig. 5a and b. The optimum pH of the solution was found at 6 for the investigated heavy metal ions. Because the metal ions can exist in their free state and their hydroxide precipitation form can be disregarded, this pH value was chosen (Fertu et al. 2022).

Due to the presence of additional H^+^ ions, which compete with the metal ions for biosorption sites, the absorption of metal ions in the acidic medium was restricted at low values of pH ˂ 4. However, at higher pH levels, the metal ion was about to hydrolyze and create metal hydroxide deposition (Qasem et al. 2021; Wallace et al. 2022). This is due to the colloidal form formation of heavy metals with OH^−^ in the aqueous media. Therefore, the quantity removed, *q*_e_, and % removal of Cd(II), Pb(II), and Zn (II) of the used biosorbent are clearly arranged at the optimum condition in the following order: Pb(II) > Cd(II) > Zn(II). It is found that Pb(II) in a single solution is the highest removal efficiency (83%) with tea pulp at pH = 6 (Pasgar et al. 2022).

#### Influence of biosorbent dose

As shown in Fig. 5c and e, with increasing the dose of the investigated biosorbents from 1.6 to 23.3 g L^−1^, the three heavy metals gradually increased the biosorption percentage. Such increment in the removal percentage of the studied metals with increment in the biosorbent dose can be associated with the more available binding sites. Afterward, the removal slowly achieved stability. Such findings are reported by Chen et al. (2022), where the modified biochar dosage was more than 0.25, 0.50, and 0.75 mg with Pb(II), Zn(II), and Cd(II), respectively. The decreasing removal percentage of Zn(II) with a further increment of biosorbent dose could be due to the effect of particle concentration, which is driven by interactions between particles. These interactions may physically deny some adsorption sites to the adsorbing solutes in a system with a higher solid content, resulting in reduced *R*%, or the electrical surface charges on the densely packed particles reduce the attractive forces between the surfaces of the biosorbent and the adsorbing solutes, resulting in electrostatic interferences (Afroze et al. 2016).

On the other hand, the uptake capacities of the metals were decreased with increasing the biosorbent doses (Fig. 5d and f). Such behavior can be explicated by Eq. (4) (Abdelfatah et al. 2022; El-Maghrabi et al. 2022; Mahmoud et al. 2022a).

4 $${q}_{e}=\left(R\times {C}_{0}/W\right)\times 100$$

Equation 4 demonstrates the inverse relation of uptake capacity to biosorbent dose. Hence, the increment of *W* causes decrement in *q*_e_ at fixed *C*_0_. Furthermore, the increment of *W* causes (1) unsaturation of biosorbent adsorption sites and (2) decrement in the biosorbent surface area due to the aggregation of the particles.

### Kinetic models

**Fig. 6:**
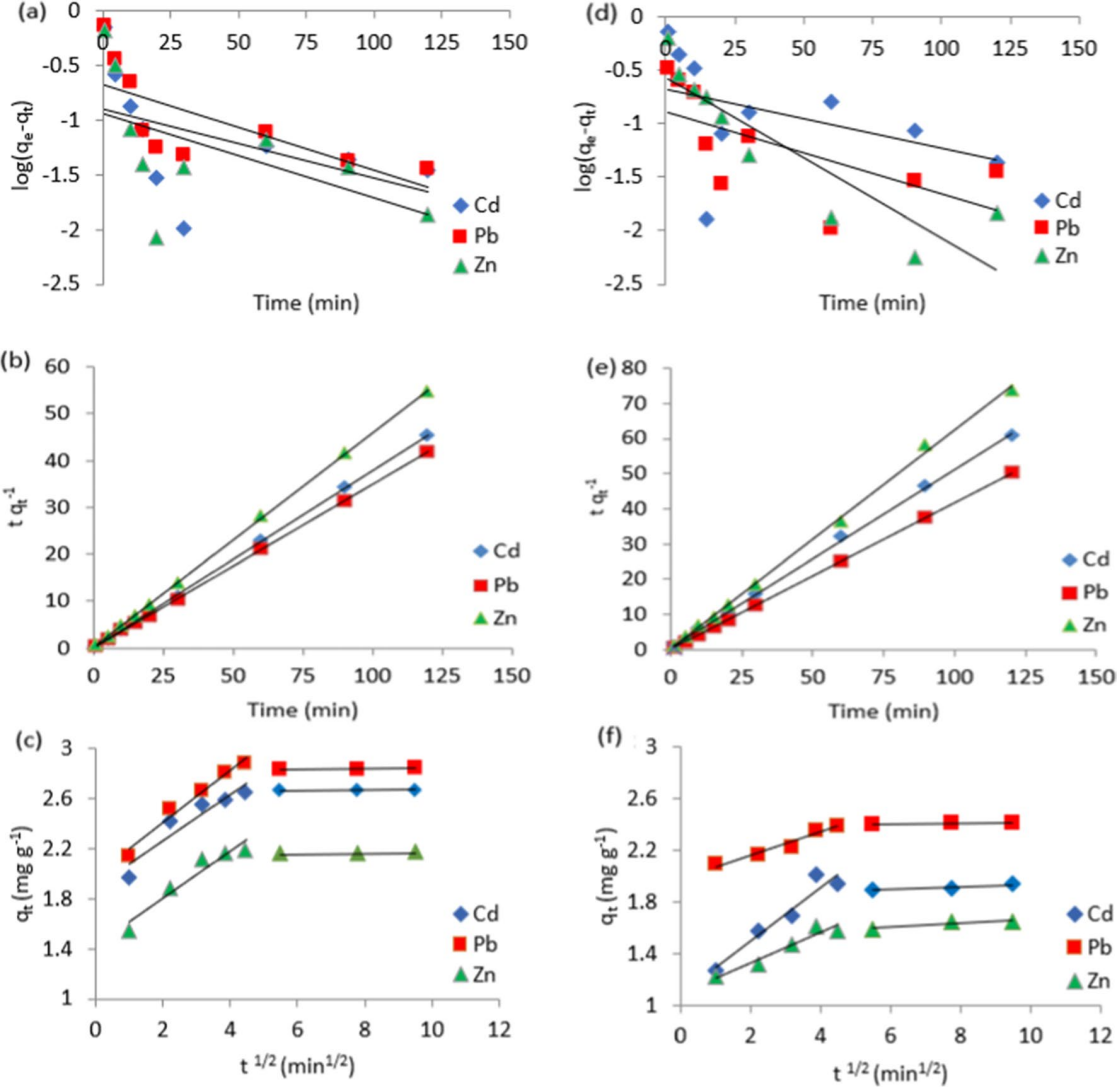
**a**, **d** Pseudo-first order, **b**, **e** pseudo-second-order and **c**, **f** intra-particle diffusion plots of Cd(II), Pb(II), and Zn(II) biosorption onto (a, b, c) cotton stalks and (d, e, f) date palm stones

Figure 6a and d shows Log (*q*_e_ − *q*_t_) against time based on Eq. S3. A straight line with a slope of the pseudo-first-order rate constant *k*_1_ and an intercept of log *q*_e_ was produced by the linear fitting. Those values, together with the linear correlation coefficient *R*^2^, are shown in Table 1. Whereas Fig. 6b and e represents linear (*t*/*q*_t_) plots against time. The calculated parameter values, including *R*^2^, are presented in Table 1. Taking into account the linear regression correlation coefficient values and how closely the experimental adsorption capacity matches the theoretical value, the best-suited model is chosen (Mahmoud et al. 2016). Therefore, the experimental results using both of the analyzed biosorbents fit and are described by the pseudo-second-order model.

**Table 1:** Experimental and calculated kinetic parameters of the investigated biosorbents

Biosorbent	Metal	*q*_e_ (exp) mg g^−1^	Pseudo-first-order model			Pseudo-second-order model		
			*k*_1_ min^−1^	*q*_e_ (calc)	*R*^2^	*k*_2_ g mg^−1^ min^−1^	*q*_e_ (calc)	*R*^2^
Cotton stalks	Cd(II)	2.67	0.015	0.13	0.240	1.40	2.65	1.0
	Pb(II)	2.88	0.018	0.21	0.501	0.86	2.86	0.999
	Zn(II)	2.19	0.018	0.12	0.291	1.06	2.18	0.999
Date palm stones	Cd(II)	2.01	0.017	0.32	0.609	0.49	1.97	0.999
	Pb(II)	2.39	0.018	0.13	0.409	1.27	2.40	0.999
	Zn(II)	1.61	0.034	0.27	0.801	1.33	1.60	0.998

The behavior of adsorption kinetics can be through surface and pore diffusions. Figure 6c and f illustrates the intra-particle fitting plots of cotton stalks and date palm stones toward the studied heavy metal ions. The first linear trend depicted the progressive adsorption stage, which was rate-limited by intra-particle diffusion. The equilibrium stage, shown by the second linear plot, was when intra-particle diffusion slowed even more as the solution concentration dropped. It is also noteworthy that the data points did not pass through the origin, indicating that other kinetic models were likely involved in the adsorption process (Tang et al. 2018).

### Isotherm study

**Fig. 7:**
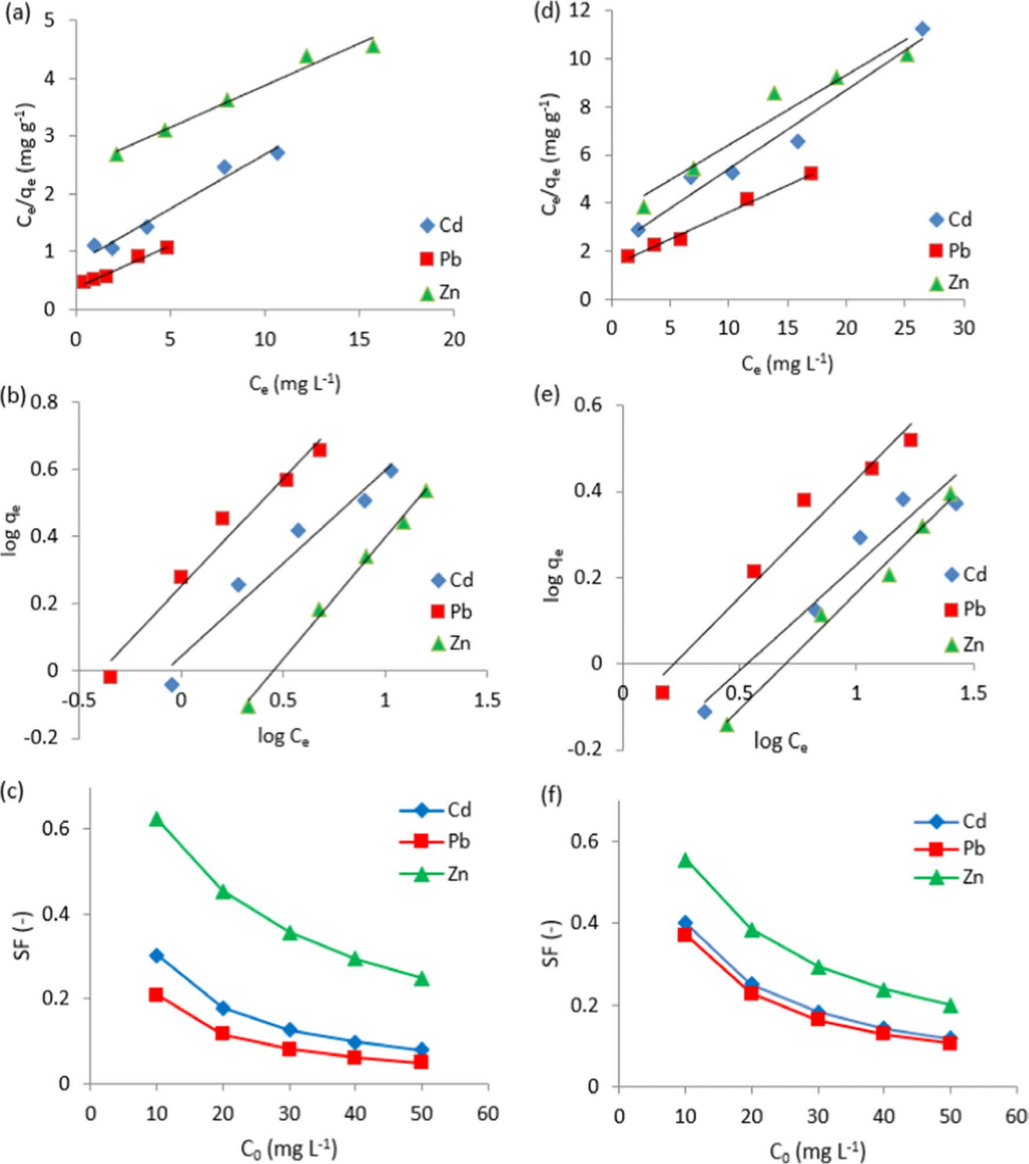
Linear **a**, **d** Langmuir isotherm, **b**, **e** Freundlich isotherm, and **c**, **f** separation factors for (a, b, c) cotton stalk and (d, e, f) date palm stones

Langmuir, Freundlich, and the separation factor have been tested to illustrate the equilibrium attributes of the adsorption (Ghaedi et al. 2013). The corresponding equilibrium parameters and the correlation coefficient of both isotherm models are given in Table 2. Langmuir’s hypothesis depends on monolayer sorption on a homogenous surface without interaction between adsorbates. Table 2 shows the superior of *q*_m_ for cotton stalks to date palm stones, which is calculated from Fig. 7a and d. The considerable shift in the carboxyl and carbonyl groups on the biosorbent surface may be the cause, besides the ion exchange as illustrated in the previous section, which augments electrostatic interactions between cotton stalks as biosorbent and the studied metal ions. The Langmuir model’s high correlation coefficient attests to its suitability for fitting experimental data.

**Table 2:** Langmuir and Freundlich isotherm parameters of the investigated biosorbents for heavy metals removal

Biosorbent	Metal	Langmuir			Freundlich		
		*q*_m_ (mg g^−1^)	*K*_L_ (L mg^−1^)	*R*^2^	*K*_f_ (mg g^−1^)	*n*	*R*^2^
Cotton stalks	Cd(II)	5.34	0.23	0.970	1.11	0.55	0.952
	Pb(II)	6.91	0.38	0.982	3.77	0.64	0.965
	Zn(II)	6.65	0.06	0.986	2.11	0.72	0.995
Date palm stones	Cd(II)	3.05	0.15	0.965	1.84	0.49	0.934
	Pb(II)	4.41	0.17	0.992	1.31	0.55	0.956
	Zn(II)	3.47	0.08	0.937	2.36	0.54	0.986

The values of *K*_L_ for Pb(II) adsorption by cotton stakes and date palm stones are higher than those values for Cd(II) and Zn(II). This indicates the more affinity of Pb(II) to the adsorption sites of cotton stakes than date palm stones.

In Freundlich isotherm (Fig. 7b and e), *n* value should range between 0 and 1, which measures the adsorption intensity. As *n* values are < 1, the adsorption isotherm is favorable (Mahmoud et al. 2021a). With the adsorption of Zn(II), Langmuir and Freundlich isotherms can be applied to both biosorbents, suggested that heterogeneous and monolayer surface conditions can both exist. The separation factor (SF) can be used to anticipate and assess the applicability of the adsorption process. Figure 7c and f proves that the adsorption isotherm is favorable because SF values were in the range of 0–1. Since the values of SF of the investigated biosorbents with Pb(II) were less than Cd(II) and Zn(II), it proves that the biosorption order of the targeted metal ions.

### Multi-metal adsorption

**Fig. 8:**
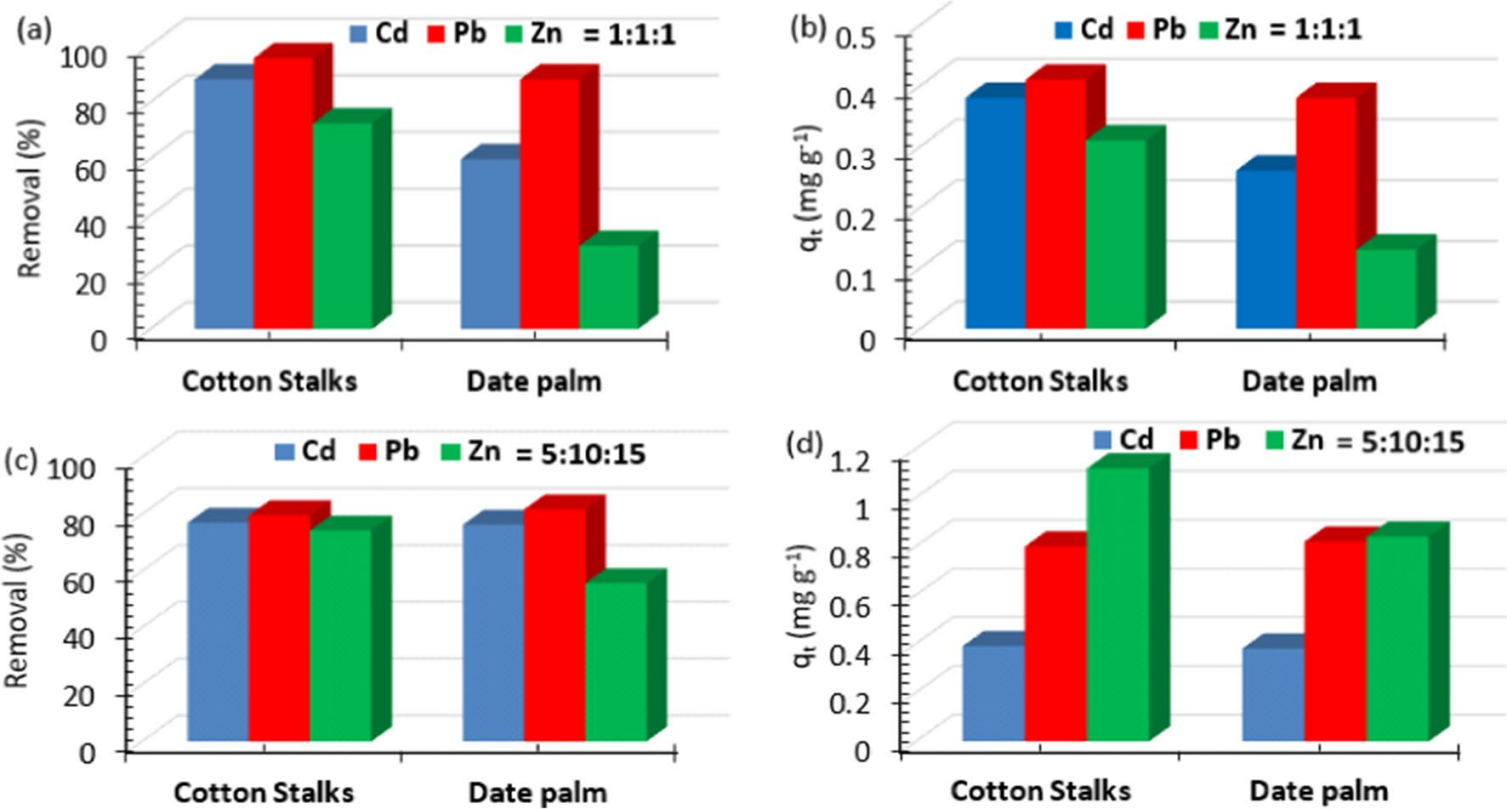
Multi-metal **a**, **c** removal and **b**, **d** uptake capacity with mass ratios of 1:1:1 and 5:10:15 for cotton stalks and palm date stones at optimum conditions. 1:1:1 ratio represents 10 mg L^−1^ for each metal and 5:10:15 ratio represents 5, 10, and 15 mg L^−1^ for Cd(II), Pb(II), and Zn(II), respectively

The tertiary adsorption of the Cd(II), Pb(II), and Zn(II) mixtures were analyzed, with mass ratios of (1:1:1) and (5:10:15) at the optimum experimental conditions. Such ratios were chosen based on the real analyzed wastewater. Figure 8 shows that the studied metal uptake capacities were decreased even if there is preferential adsorption for specific metal ion, such as Pb(II), there is competition between the metal ions for the same adsorption surface locations. The adsorption procedure is impacted by the physical and chemical characteristics of the investigated metal ions. For example, the high electronegativity and large ion radius of Pb(II) cause the inhibition of the adsorption of the coexisted metal ions (Cd(II) and Zn(II)) (Deng et al. 2019; Kajeiou et al. 2020; Mahamadi 2019).

### Mechanism of biosorption

Based on the experimental and modeling results, the finding selectivity can be explained by the solute’s relative affinity for the liquid and solid phases. The distribution of functional groups on the biosorbents from FT-IR, the differences in metal ionic sizes, and the interaction of those ions with the biosorbents can explain how this affinity emerges (Chen et al. 2021; Mahmoud et al. 2021a): (i) The ionic radius of the metal ions are 1.19 Å, 0.95 Å, and 0.74 Å for Pb(II), Cd(II), and Zn(II), respectively. Pb(II) had the potential for becoming the most adsorbed since it has the largest ionic radius. As a result, surface biosorption has a more significant impact on the biosorption capacity than microporous biosorption. (ii) In comparison to Cd(II) and Zn(II), whose respective hydrated radii are 0.426 and 0.430 nm, Pb(II) has a smaller hydrated radius (0.401 nm). (iii) In comparison to Cd(II) (1.69) and Zn(II), Pb(II) has a higher electronegativity of 2.10 (1.65). (iv) The larger unhydrated ions have a lower charge density and less grip on the hydration water, which results in a weaker link between the metal ion and water phase.

Physical adsorption could be happened due to a weak Van der Waal’s force. The presence of carbonyl/carboxyl and hydroxyl groups on the biosorbents has a role in the binding of heavy metallic cations, as reported in Esfandian et al. (2023). The adsorption mechanism could be (i) surface complexation where reactions occurred at the surface functional group X-OH (Mahmoud et al. 2016); (ii) ion exchange where K and Ca disappeared after the biosorption process, as depicted in EDX results; (iii) pore filling, where surface and film diffusion adsorption has a role in the uptake process as shown in Fig. 6c and f. As reported by Imran-Shaukat et al. (2022) several physicochemical processes are involved in the adsorption of heavy metal ions by agricultural waste-based biosorbents, involving ion exchange, complexation, physisorption, and intra-particle diffusion.

In addition, each metal ion behavior could be attributed to the mutual and varied inhibitory effects on the sorption of the metal ions in ternary solutions. Equation (5) is utilized to designate the synergistic and antagonistic behavior of the biosorption multi-metal solutions.

5 $${R}_{sa}=\left({q}_{e,x}/{q}_{e,y}\right)\times 100$$

where *q*_e,x_ and *q*_e,y_ denote the uptake capacity of a metal ion in multi-metal solutions and mono-metal solution, respectively. *R*_sa_ denotes non-interactive behavior when equals to 100. Our findings revealed that all calculated *R*_sa_ percentages are ˂ 100 (Fig. S1), which denote antagonistic behavior of the interaction between the multi-metal solutions.

### Real wastewater analysis

**Fig. 9:**
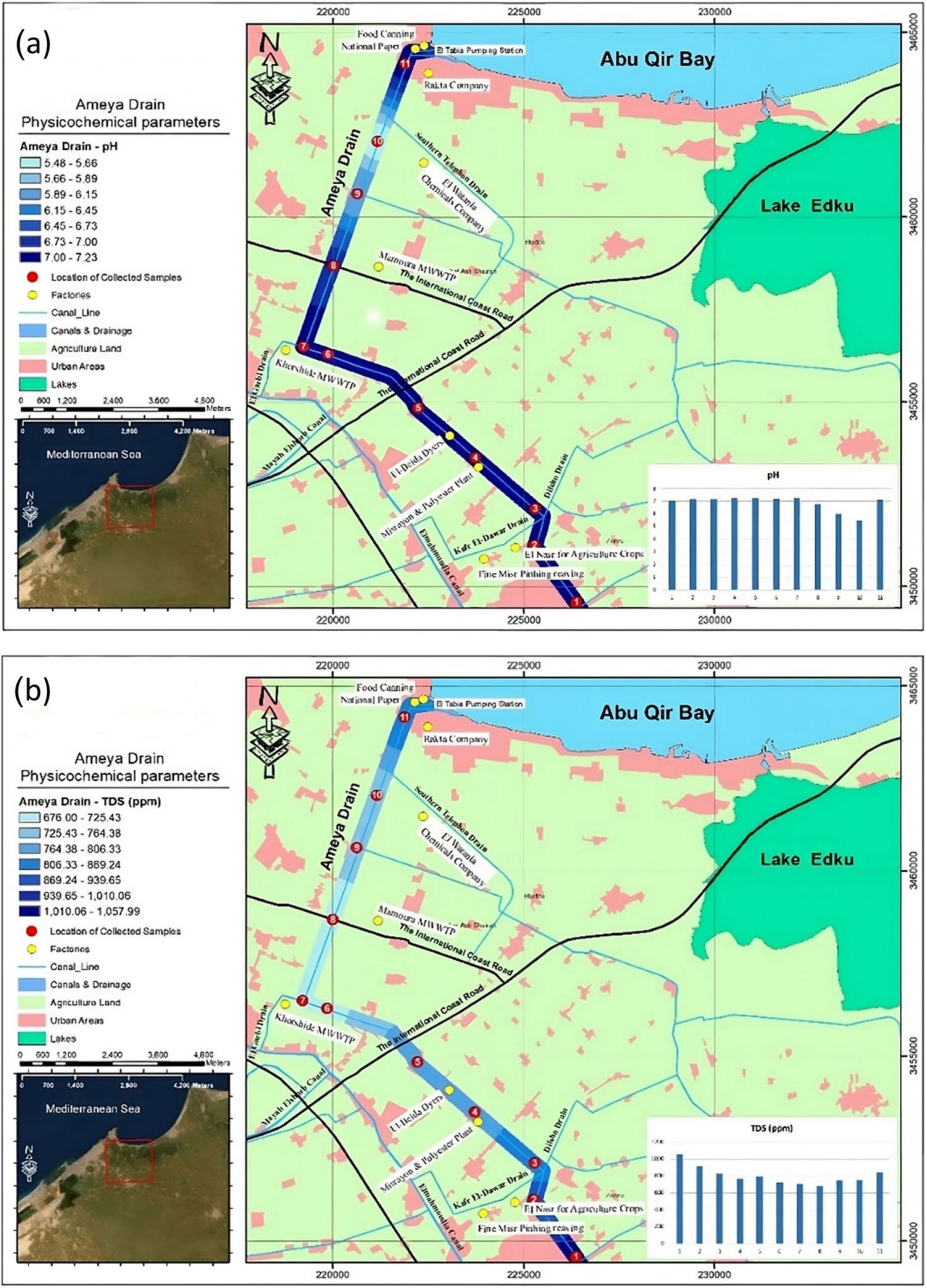
**a** The distribution of the measured pH values and **b** the distribution of the measured TDS concentrations along EL-Ameya Drain

Physical parameters of the collected wastewater from EL-Ameya Drain were analyzed as shown in Fig. 9a,b. pH and TDS were recorded in the range of 5.48–7.23 and 676–1058 mg L^−1^, respectively. Additionally, as shown in Table 3, composite wastewater samples from the same areas throughout four distinct seasons were collected and tested for Cd, Pb, and Zn.

**Table 3:** Analysis wastewater samples collected from EL-Ameya Drain in Egypt in different seasons

Collection seasons	Concentration of the detected metal ions Before biosorption (mg L^−1^)			Concentration of the detected metal ions After biosorption (mg L^−1^)		
	Cd(II)	Pb(II)	Zn(II)	Cd(II)	Pb(II)	Zn(II)
Autumn 2021	0.038 ± 0.005	0.581 ± 0.025	0.908 ± 0.012	Not detected	Not detected	Not detected
Winter 2021	0.009 ± 0.001	0.520 ± 0.037	0.835 ± 0.031	Not detected	Not detected	Not detected
Spring 2022	0.004 ± 0.001	0.095 ± 0.033	0.322 ± 0.011	Not detected	Not detected	Not detected
Summer 2022	0.005 ± 0.001	0.371 ± 0.024	0.720 ± 0.019	Not detected	Not detected	Not detected

As suggested by batch trials, biosorption tests were carried out on real wastewater samples that had been collected under optimum conditions. The applied biosorbents, either cotton stalks or date palm stones, succeeded to adsorb the metal ions traces in the wastewater and not detected by atomic absorption spectrometer (AAS) with detection limits of Pb; 0.085 µg L^−1^, Cd; 0.007 µg L^−1^, and Zn; 0.008 µg L^−1^. All samples were measured in triplicates. After applying either cotton stalks or date palm stones as adsorbents for the treatment of the collected wastewater with the optimum conditions. Almost complete removal of the investigated heavy metals was obtained as shown in Table 3.

### Regeneration of biosorbents

**Fig. 10:**
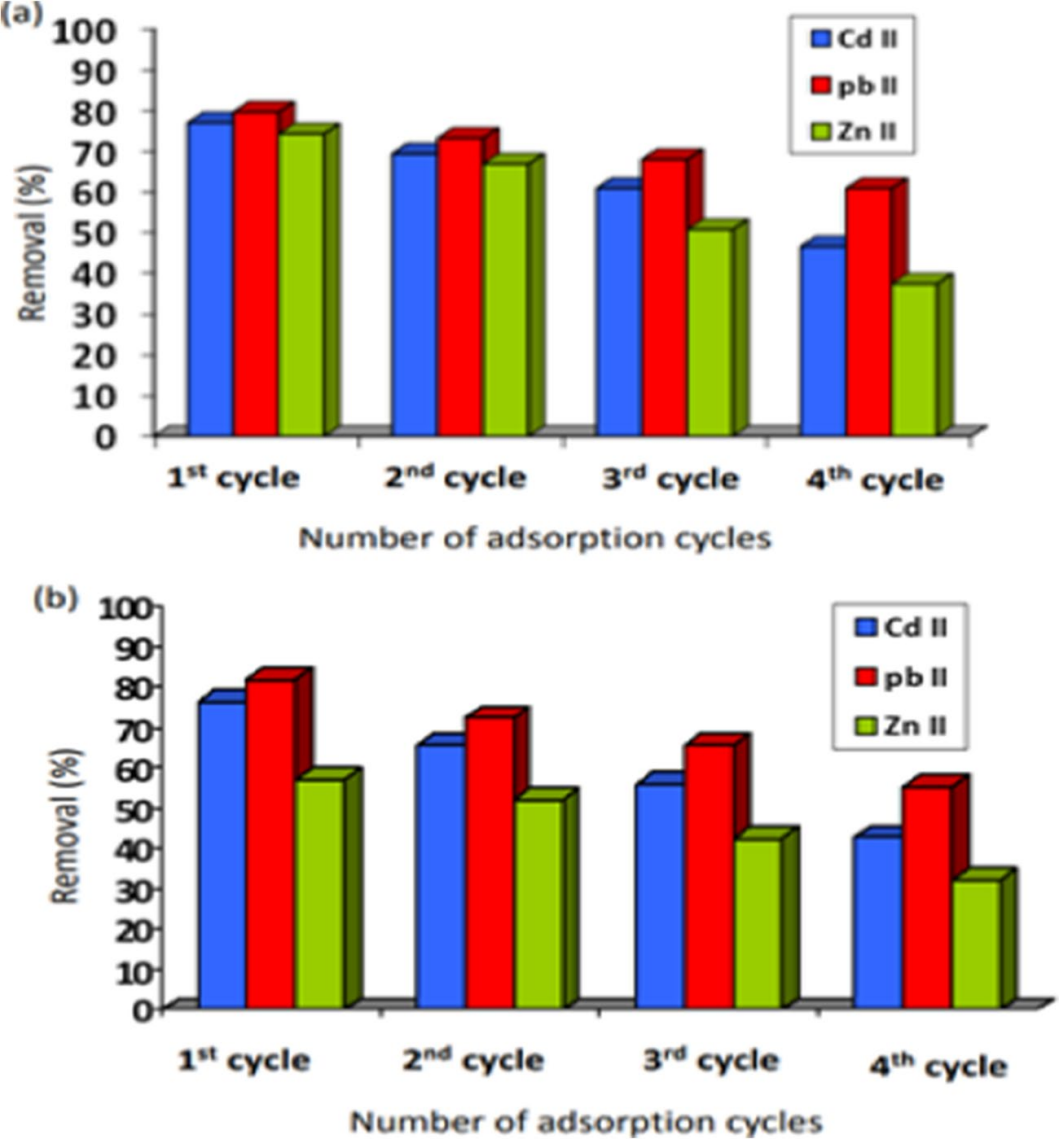
Adsorption-desorption cycles of Cd(II), Pb(II), and Zn(II) from multi-metal solution by the investigated **a** cotton stalk and **b** palm date biosorbent

Reusing biosorbents could be considered an integral component of circular economy. One of the prerequisites of circular economy is to overcome current barriers to water reuse. Regeneration of biosorbents is considered a valuable source of extracted materials. Desorption studies were conducted with 0.2 M HCl for four successive cycles. Figure 10 shows a gradual decrease in Cd(II), Pb(II), and Zn(II) removal efficiencies from multi-metal solution with an increasing number of cycles. For cotton stalks with Cd (II), the removal % of the four successive cycles were found to be 77.1%, 69.5%, 61.0%, and 46.6%, respectively. Quite similar behavior was detected with date palm stones where the four successive cycles were found to be 76.4%, 65.6%, 56.1%, and 42.7%, respectively. For cotton stalks with Pb(II), the removal % of the four successive cycles were found to be 79.7%, 73.3%, 68.1%, and 61%, respectively. While 76.4%, 65.6%, 56.1%, and 42.7%, respectively, for palm dates. For Zn(II), the removal % of the four successive cycles were found to be 74.4%, 67.0%, 50.8%, and 37.5%, respectively, with cotton stalk. However, the removal % of the four successive cycles with palm dates were found to be 57.1%, 52.2%, 42.6%, and 32.1%, respectively.

The loss of its adsorption active sites at low pH levels may account for the declining adsorption capability throughout regeneration cycles. It is noticed that the biomass of cotton stalk could be recycled up to 3 times for Cd(II), 4 times for Pb(II), and 2 times for Zn(II) ions with accepted efficiency loss (Zhang et al. 2020).

A significant portion of the metal ions adsorbed and turned into irreversible sorption, and the biosorbent load capacity is lost for the repeated adsorption-desorption cycles, according to the minor decline in removal efficiency after each cycle. The sequence of desorption was Pb(II), Cd(II), and Zn (II). This results from a more efficient interchange of Zn(II) with the ions present in the surface functional groups of the biomass during the adsorption process. Pb(II) ions are easier to desorb because, due to their bigger size, they do not permeate pores and channels to the same extent as Zn(II) ions. The desorption efficiency rises with increasing ionic radii (Panek et al. 2021).

Table S2 illustrates the biosorption efficiency and capacity of the studied heavy metals compared to literature. As it is reported in Table S2, the biosorption efficiency and capacity for Pb(II), Cd(II), and Zn(II) are comparable with other biosorbents. Even it is better than reported by Çelebi et al. (2020) who found the maximum biosorption capacity derived from Langmuir model as 2.468 mg g^−1^ for Cd(II), 1.457 mg g^−1^ for Zn(II), and 1.197 mg g^−1^ for Pb(II) as well as Mahmoud et al. (2021d) and Ligarda-Samanez et al. (2022).

## Conclusions

Adsorption process using cotton stalks and date palm stones demonstrated optimum mono- and multi-metal removal of the studied heavy metals at pH = 6. Adsorption pattern followed the order: Pb(II) ˃ Cd(II) ˃ Zn(II). The fitted kinetic model was pseudo-second-order with *R*^2^ > 0.99 along with the intra-particle diffusion model to prove that adsorption is a rate-limited process. The adsorption isotherm is favorable because separation factor values were in the range of 0–1. The competition between the targeted metal ions was linked to the decrease in removal of multi-metal solutions compared to mono-metal ones. This highlights the significance of investigating the biosorption efficiencies and capacities of biosorbents with other coexisting heavy metals. The calculated *R*_sa_ confirmed the antagonistic behavior of the interaction between the multi-metal solutions at optimum conditions. In the real wastewater application, cotton stalks and date palm dates were found to be effective in removing heavy metal ions completely. Successful four adsorption-desorption cycles were conducted. The possibility of biosorbent regeneration with several cycles gives a good opportunity for metals recovery in the aspects of circular economy. In view of their availability, cotton stalks and date palm stones could be considered excellent candidates for the removal of heavy metals from wastewater.

### Supplementary information

Below is the link to the electronic supplementary material.

Supplementary file1 (PDF 150 KB)

## Data Availability

The data that support the findings of this study are available from the corresponding author upon reasonable request.
