# Correction to: Potentials of mono‑ and multi‑metal ion removal from water with cotton stalks and date palm stone residuals

**DOI:** 10.1007/s11356-023-27571-4

**Published:** 2023-05-12

**Authors:** Heba Nagy, Manal Fawzy, Elsayed Hafez, Alaa El Din Mahmoud

**Affiliations:** 1 Environmental Sciences Department, Faculty of Science, Alexandria University, Alexandria, 21511 Egypt; 2 Green Technology Group, Faculty of Science, Alexandria University, Alexandria, 21511 Egypt; 3 National Biotechnology Network of Expertise (NBNE), Academy of Scientific Research and Technology (ASRT), Cairo, Egypt; 4 Plant Protection and Biomolecular Diagnosis, Arid Lands Cultivation Research Institute (ALCRI), City of Scientific Research and Technological Applications (SRTA-City), Alexandria, 21934 Egypt

**Correction to: Environmental Science and Pollution Research**

10.1007/s11356-023-27137-4

The line numbers were included in the published proof.

The Original article has been corrected.

