# Supplementary material for: Potentials of mono- and multi-metal ion removal from water with cotton stalks and date palm stone residuals
Source: Environ Sci Pollut Res Int. 2023 May 1;31(28):39849–65. doi: 10.1007/s11356-023-27137-4 (PMC11189956; doi:10.1007/s11356-023-27137-4)
Supplement: Supplementary file 1 — Supplementary file1 (PDF 150 KB) [file 11356_2023_27137_MOESM1_ESM.pdf]

Supplementary information

Potentials of mono- and multi-metal ions removal from water with cotton stalks and date palm stones residuals

Heba Nagy[1], Manal Fawzy[1,2,3], Elsayed Hafez [4], Alaa El Din Mahmoud[1,2*]

[1]Environmental Sciences Department, Faculty of Science, Alexandria University, 21511 Alexandria, Egypt.
[2] Green Technology Group, Faculty of Science, Alexandria University, 21511 Alexandria, Egypt
[3]National Biotechnology Network of Expertise (NBNE), Academy of Scientific Research and Technology (ASRT), Egypt.
[4]Plant Protection and Biomolecular Diagnosis, Arid Lands Cultivation Research Institute (ALCRI), City of Scientific Research and Technological Applications (SRTA-City), 21934 Alexandria, Egypt.

***Corresponding author:** Dr. Alaa El Din Mahmoud. Email: alaa-mahmoud@alexu.edu.eg

Materials and methods

Characterization of biosorbents

The surface morphologies of the samples were examined using a scanning electron microscope (SEM; JOEL-JSM-IT200, Japan). Using an ion sputter evaporator, gold was deposited on the prepared SEM samples. The elemental compositions of the investigated biosorbents before and after the treatment process were measured using Energy dispersive X-ray spectroscopy (EDX). To assess the possible involvement of different functional groups of the investigated biosorbents in the biosorption of heavy metals, FT-IR spectrum was conducted over the range 4000 - 800 cm^{-1} using Cary 630 (Agilent Technologies, Germany).

Evaluation of biosorption experiments

The uptake capacity and removal percentage of Cd(II), Pb(II) or Zn(II):

$$q_e = (C_0 - C_e)\ V/W\ , \qquad (S1)$$

$$R= [(C_0 - C_e)/ C_0]\ x100\ , \qquad (S2)$$

where, q_e the quantity of solute adsorbed per unit weight of biosorbent (mg g^{-1}). C_0 and C_e: are the initial and final metal ions concentration (mg L^{-1}), respectively. V and W: are the solution volume (L) and the biosorbent weight (g), respectively. R: is the metal ions removal efficiency (%).

Kinetics models were used to examine the controlling mechanism of biosorption process such as Pseudo 1st order model (eq. S3), Pseudo 2nd order model (eq. S4), and intra-particle diffusion (eq. S5).

$$\log (q_e-q_t) = \log q_e - (K_1/2.303)\, t , \qquad (S3)$$

$$t/q_t = 1/ K_2 q_e^2 + (1/q_e)\, t , \qquad (S4)$$

$$q_t = (K_{id} \times t_{1/2}) + C_i, \qquad (S5)$$

where; q_e and q_t: are the amount of metal ions adsorbed at equilibrium time and at time t (mg g^{-1}), respectively. K_1 and K_2: are the pseudo-first-order rate constant (min^{-1}) and the rate constant of pseudo-second-order kinetics (g mg^{-1} min^{-1}), respectively. K_{id}: is the intra-particle diffusion rate constant (mg g^{-1} $min^{-1/2}$), and C_i: is the intercept.

Different models describe the equilibrium between the adsorbate and biosorbent, among these models Langmuir and Freundlich isotherms. The Langmuir biosorption isotherm is often used to describe the maximum biosorption capacity of biosorbent. It is given by eq. 6, whereas Freundlich isotherm is expressed by eq. 7. Moreover, the separation factor (SF) is calculated to indicate the feasibility of the adsorption process (eq. 8).

$$C_e/q_e = 1/ K_L q_m + C_e/q_m , \qquad (S6)$$

$$\mathrm{Log}(q_e) = \log(K_f) + n \log(C_e) , \qquad (S7)$$

$$SF = 1 / (1+K_L C_0) , \qquad (S8)$$

where, q_m: is the maximum biosorption uptake under the given condition (mg g^{-1}). K_L: is the coefficient related to the affinity between the sorbent and sorbate (L mg^{-1}). K_f: is the Freundlich adsorption constant, which characterizes the strength of adsorption [(mg g^{-1}) (mg L^{-1})^n]. n: is the measure of adsorption intensity. SF: is the separation factor (dimensionless).

Regeneration of biosorbents

To conduct the regeneration investigation of the studied biosorbents, 10 g L^{-1} of each biosorbent was added into Erlenmeyer flasks containing 100 mL of 5, 10, and 15 mg L^{-1} of Cd(II), Pb(II), and Zn(II), respectively. After equilibration for 30 min under room temperature, the adsorbent was recovered, and the adsorption capacity determined. By washing the used biosorbent surface three times in a row with distilled water, followed by a 60 °C oven dry to constant mass, residual metal ions were removed.

Using 0.2 M HCl, the metal ions were desorbed. The recovered adsorbent was mixed in 30 mL of the desorption agent for 30 min at 150 rpm. The adsorbate–adsorbent mixtures were filtered using Whatman no 1 filter paper. The concentrations of the Cd(II), Pb(II) and Zn(II) ions were determined using Atomic Absorption Spectrometer and the desorption percentage was computed using eq. 1.

$$\text{Desorption efficiency} = (q_{des} / q_{ads}) \times 100 , \qquad (1)$$

where q_{des}: is the amount of metal left on the biomass after the desorption process. q_{ads}: is metal ion uptake capacity.

The recovered biosorbents were dried at 60 °C, and reused in further adsorption–desorption cycles to determine the reusability of the biomass. The reusability of the biomass was conducted in four successive adsorption–desorption cycles.

Table S1. FTIR Characterization of the investigated date palm and cotton stalk stems before and after biosorption of Cd (II), Pb (II) and Zn (II) ions

Biosorbent	Peak	Transmission band (cm^{-1})						Assignment
		Cd (II)		Pb (II)		Zn (II)		
		Before	After	Before	After	Before	After	

**Date palm**	**1**	3322.9	3290	3322.9	3262.1	3322.9	3283.5	stretching vibration of aliphatic OH-group
**Cotton stalk**	**1**	3337.1	3324.7	3337.1	3328.9	3337.1	3332.1	
**Date palm**	2	2921.6	2922.4	2921.6	2921.7	2921.6	2921.8	stretching vibration of aliphatic CH-group
**Cotton stalk**	2	2917.2	2913.7	2917.2	2913.8	2917.2	---------	
**Date palm**	**3**	1733.7	---------	1733.7	---------	1733.7	---------	C=O group in non-ionic carbonyl group
**Cotton stalk**	3	1733.7	1734.2	1733.7	1733.5	1733.7	1733.8	
**Date palm**	**4**	1653.0	---------	1653.0	---------	1653.0	---------	Asymmetric Stretching of -COO- in ionic carboxylic group
**Cotton stalk**	**4**	1644.9	---------	1644.9	1653.1	1644.9	1653.3	
**Date palm**	**5**	1457.1	---------	1457.1	---------	1457.1	---------	Carboxylate group -COO-symmetric stretching
**Cotton stalk**	**5**	1457.1	1457.6	1457.1	1457.3	1457.1	1457.5	
**Date palm**	**6**	1238.4	1238.8	1238.4	1237.8	1238.4	1237.8	C-O Sterching
**Cotton stalk**	**6**	1235.3	1234.5	1235.3	1235.3	1235.3	1234.1	

Mechanism of biosorption

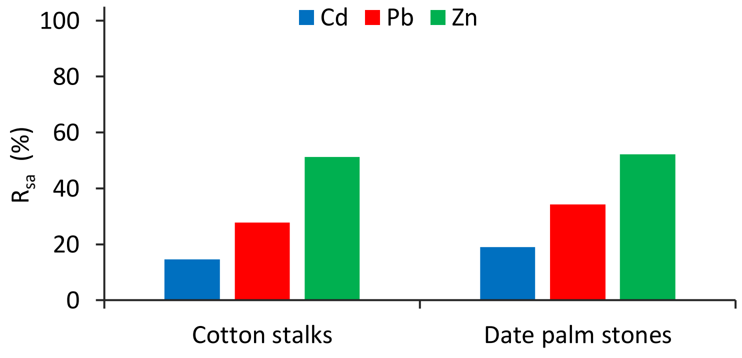

Fig. S1 The quantitative analysis of metal ions uptake in multi-metal solutions.

Table S2 Biosorption capacity/efficiency (mg g^{-1} / %) of heavy metals by different biosorbents.

**Biosorbent**	**Biosorption capacity/efficiency (mg g^{-1} / %)**	**References**
Papaya wood	Cd(II); 97.8% > Cu(II); 94.9% > Zn(II); 66.8%	(Saeed et al. 2005)
Green coconut shell (powder)	Cr(III); 90% > Cr(VI); 86% > Cd(II); 99%	(Pino et al. 2006b)
Typha domingensis biomass	Cd(II); 78.1% > Ni(II); 31.0%	(Fawzy et al. 2016)
Flax fibers	Cu(II); 112 > Pb(II); 122 > Zn(II); 71 mmol kg^{-1}	(Kajeiou et al. 2020)
Bacterial strain isolated from River Yamuna	Cu(II); 88.45% > Ni(II); 82.45% > Zn(II); 69.99% > Cd(II); 63.04% > Cr(VI); 48.93%	(Sodhi et al. 2020)
Agave Bagasse	Pb(II); 93.14 > Cd(II); 28.50 > Zn(II); 24.66 mg g^{-1}	(Cholico-González et al. 2020)
Brewed tea waste	Pb(II); 97.97% > Ni(II); 82% > Zn(II); 76% > Cd(II); 84.74%<br>Cd(II); 2.468 mg g^{-1}, Zn(II);1.457 mg g^{-1}, Pb(II);1.197 mg g^{-1}, Ni(II); 1.163 mg g^{-1}	(Çelebi et al. 2020)
Treated olive mill solid residue	Pb(II); 4.58 > Cd(II); 4.52 > Cu(II); 4.367 mg g^{-1}	(Mahmoud et al. 2021d)
Waste rubber tires	Cu(II); 12.43 > Pb(II); 9.68 > Zn(II); 4.99 mg g^{-1}	(Cherono et al. 2021)
Modified Polymeric Biosorbents from Rumex acetosella	Pb(II); 96.14% > Zn(II); 36.30% > Cd(II); 34.10% > As 32.50%	(Ligarda-Samanez et al. 2022)
cotton stalks and date palm	Pb(II); 98% > Cd(II); 92.1% > Zn(II); 78.9%<br>& Pb(II); 4.41-6.91 mg g^{-1}<br>Cd(II); 3.05-5.34 mg g^{-1}<br>Zn(II); 3.47-6.65 mg g^{-1}	This work
